# Editorial: Hybrids Part B: Hybrids for Drug Delivery

**DOI:** 10.3389/fbioe.2021.720714

**Published:** 2021-06-30

**Authors:** Wei Li, Kai Zheng, Vilma Petrikaite

**Affiliations:** ^1^Drug Research Program, Division of Pharmaceutical Chemistry and Technology, Faculty of Pharmacy, University of Helsinki, Helsinki, Finland; ^2^Institute of Biomaterials, University of Erlangen-Nuremberg, Erlangen, Germany; ^3^Laboratory of Drug Targets Histopathology, Institute of Cardiology, Lithuanian University of Health Sciences, Kaunas, Lithuania

**Keywords:** hybrids, drug delivery, controlled release, nanoparticles, nanomedicines

Hybrids, consisting of two or more components at the nanometer or molecular level, have recently emerged as novel biomaterials for drug delivery applications. Hybrids usually show characteristics in between the original components, but also may exhibit new and unique physical, chemical, and/or biological properties not possessed by the original components. Hybrid systems could combine the benefits of different structural components to synergize the outcome of drug delivery for therapeutic applications. Many attempts have been dedicated to design and synthesize novel hybrid biomaterials with several concurrent features, such as favorable size/shape for efficient drug loading and controlled drug release, desired surface properties for targeting specific organs/tissues/cells, and satisfactory biocompatibility and biodegradation rate for clinical translation.

This Research Topic invited researchers to contribute studies on recent advances in hybrids for drug delivery. We have collected three review articles and one original research article, which highlight several emerging trends of hybrids for drug delivery.

Xu et al. in their publication present a synthesis, *in vitro* release study, cytotoxicity analysis, and *in vivo* immune response examination of fabricated organic–inorganic hybrid nanoparticles used as the carriers of zoledronic acid. Those nanorods are intended to be used for osteosarcoma therapy. They consist of hyaluronic acid/polyethylene glycol polymer shell and a nano-hydroxyapatite core with zolendronic acid on it. The authors demonstrated that the compact and stable structure could achieve high drug loading efficiency, sustained drug release, and favorable biocompatibility. *In vitro* and *in vivo* experiments revealed the low cytotoxicity and acceptable immune response under low-dose nanoparticle treatment.

Cai et al. review the recent progress in the synthesis and biomedical applications of hybrid hydrogels. Particularly, novel approaches for fabricating micro/nano structure on hydrogels, including click reaction implementation, 3D printing and photopatterning, are introduced. In this review article, the authors also demonstrate the great potential of hybrid hydrogels in cancer therapeutic delivery, drug discovery, and regenerative medicine.

Yao et al. give an overview about the application and development of hybrid nanoparticles as drug carriers, and discuss the roles of nanoparticles and hybrid nanoparticles for drug delivery in chemotherapy, targeted therapy, and immunotherapy and describe the targeting mechanism of nanoparticle-based drug delivery as well as its function on reversing drug resistance.

Huang et al. summarize the recent advances of hybrids or nanomaterials designed for targeted therapy of kidney diseases and discuss the great potential of hybrids or nanomaterials for the delivery of immunosuppressants like tacrolimus and triptolide, antioxidants, or siRNAs. In this review article, the authors also highlight the achieved superior therapeutic effects and reduced systemic side effects by hybrids or nanomaterials in kidney disease animal models.

In summary, this Research Topic covers recent advances in the hybrids for drug delivery, providing new aspects of the roles of hybrids in delivering drugs for enhancing the therapeutic effects and reducing the side effects. The editors hope that the Research Topic “Hybrids Part B: Hybrids for Drug Delivery” will contribute to the progress of research and development activities in the field of hybrids for drug delivery, inspiring future work leading to the expansion of the biomedical applications of hybrids.

## Author Contributions

All authors listed have made a substantial, direct and intellectual contribution to the work, and approved it for publication.

## Conflict of Interest

The authors declare that the research was conducted in the absence of any commercial or financial relationships that could be construed as a potential conflict of interest.

